# Assessing the relationship between gout and the risk of cataract in community-dwelling older adults: mediation and moderation analysis

**DOI:** 10.3389/fmed.2025.1740517

**Published:** 2026-01-13

**Authors:** Shuilian Chen, Chunxin Lai, Fulong Luo, Yongyi Niu, Yongjie Qin, Yanlei Chen, Zhuoting Zhu, Xianwen Shang, Xueli Zhang, Yu Huang, Hongyang Zhang

**Affiliations:** 1 Guangdong Eye Institute, Department of Ophthalmology, Guangdong Provincial People’s Hospital, Guangdong Academy of Medical Sciences, Southern Medical University, Guangzhou, China; 2 Shantou University Medical College, Shantou, China; 3 Guangdong Cardiovascular Institute, Guangdong Provincial People’s Hospital, Guangdong Academy of Medical Sciences, Guangzhou, China; 4 Centre for Eye Research Australia, Melbourne, VIC, Australia; 5 Division of Population Health and Genomics, Ninewells Hospital and Medical School, University of Dundee, Dundee, United Kingdom; 6 Department of Ophthalmology, Nanfang Hospital, Southern Medical University, Guangzhou, China

**Keywords:** cataract, cohort study, glucocorticoids, gout, mediation and moderation

## Abstract

**Objective:**

This study aimed to assess whether gout is a risk for cataract and identify important factors contributing to the association.

**Methods:**

A total of 381,402 individuals from the UK Biobank were enrolled at baseline (2006–2010). Cataract was ascertained using hospital inpatient and self-reported data until early 2021. Gout was determined by ICD-9, ICD-10, self-report, and medication at baseline. The Cox regression model was used to estimate the hazard ratio (HR) and 95% confidence intervals (CIs) for the risk of cataract.

**Results:**

The risk of cataract was significantly increased in patients with gout (HR, 1.69; 95% CI, 1.48–1.94; p < 0.001), and this association was attenuated but remained significant after additional adjusting for other covariates (HR, 1.14; 95% CI, 1.04–1.26; p = 0.006). In addition, we observed a significant interaction effect between gout and glucocorticoids (GCC) use for senile cataract (p = 0.04). In the sensitivity analysis, we stratified the population by sex, diabetes mellitus (DM), and GCC use. We noticed that gout remains a risk factor for cataract in both sexes and in patients with or without GCC use. Finally, we tested the mediation effect of GCC; we observed that 2.4% of the effect between gout and cataract and 3% of the effect between gout and senile cataract was mediated by GCC use.

**Conclusion:**

This cohort study found that gout was an independent risk factor for cataract, with a significant impact on senile cataract. GCC use modified the effect of gout on the development of senile cataract (interaction effect), thus prescribing GCC to patients with gout should be actively monitored for cataract development.

## Introduction

Gout, characterized by elevated levels of uric acid and the deposition of monosodium urate crystals in joints, affects over 7 million people worldwide annually [1, 2]. Previous studies have reported that, in addition to causing excruciating arthritic pain, gout is associated with chronic kidney disease, hypertension, hyperlipidemia, coronary heart disease, and acute cerebrovascular disease [3–5]. Except for systemic conditions, patients with gout may also suffer from ocular dysfunctions, such as cataract [6]. Cataract represents a significant public health and economic burden globally, being the primary cause of visual impairment worldwide [7, 8]. Despite cataract surgery being the most prevalent surgical procedure across medical fields, there remains a high prevalence of blindness due to inadequate surgical equipment, severe surgical complications, and high surgical costs in low- and middle-income countries [9–11]. Therefore, it is imperative to identify modifiable risk factors for cataracts and establish effective treatments to slow down the progression of vision loss and alleviate the medical burden associated with the condition.

Common risk factors for cataracts include aging, smoking, alcohol consumption, trauma, surgery, tumors, and metabolic disorders. Extensive research has been conducted to investigate the association between metabolic disorders, such as hyperlipidemia or hyperglycemia, and cataract development [12–14]. However, the relationship between gout, the most prevalent hyperuricemia metabolic disorder, and cataract development remains unclear.

Although the findings are inconsistent, according to Zubenko et.al [15], a history of gout emerged as one of the strongest risk factors for age-related cataract, while Mukesh et.al could not find the association between gout and cortical, nucleus, or posterior subcapsular (PSC) cataract after adjusting for risk factors [16]. Besides, Li et al. reported that patients with gout might have a higher risk of cataract, potentially due to the intake of gout medication [17]. Considering that gout may have an impact on the incidence of cataract, a thorough and systematic investigation of the gout in cataract development is urgently required and may shed light on its etiology, providing a new treatment strategy for the prevention of cataract.

In this study, we sought to examine the association between gout and the incidence of cataract using the large-scale longitudinal cohort from the UK Biobank data. Specifically, we further investigated the mediation effect of gout medication on age-related cataract in order to demonstrate the potential mechanism or clinical intervention between gout and cataract. Specifically, glucocorticoid (GCC, one type of the first-line drug for gout) [18, 19] use was taken into account, which was an important risk factor for cataracts, and GCCs were widely used in multimorbidity among the elderly population. Therefore, we analyzed the interaction effect of GCC use as well as tested its mediation role between gout and cataract.

## Materials and methods

### Patients

The UK Biobank is a large prospective observational study, recruiting over 500,000 participants aged 40 to 73 years at enrollment [20]. Participants who were registered with the National Health Service and lived within 25 miles of 1 of 22 assessment centers throughout the United Kingdom from 2006 to 2010 were invited to the study (5.47% response rate) [21]. Baseline examinations consisting of questionnaires and enhanced assessments have been reported previously elsewhere [22]. Eye data collection began in late 2009, including additional enhancements to baseline assessment visits. Briefly, participants responded to a detailed touchscreen questionnaire that included demographic, socioeconomic, lifestyle, and systemic and eye disease information, including their cataract status. The Townsend deprivation index was included in the model because social deprivation was associated with the incidence and prevalence of gout and frequent gout attacks [23, 24].

### Ascertainment of gout

Gout was determined according to the information from self-report (Data-Field 20002, code: 1466), International Classification of Disease-10 (ICD-10, codes: M10) and ICD-9 (codes: 274) and baseline self-reported medication history (Data-Field 20003, code ‘1140875408’ for allopurinol, ‘1140875490’ for probenecid, ‘1140909890’ for sulfinpyrazone, ‘1140875486’ for colchicine) from the Hospital Episode Statistics (England), the Scottish Morbidity Record (Scotland), and the Patient Episode Database (Wales). Finally, we defined baseline gout as a diagnosis <1 year since recruitment.

### Ascertainment of cataract

**Figure 1:**
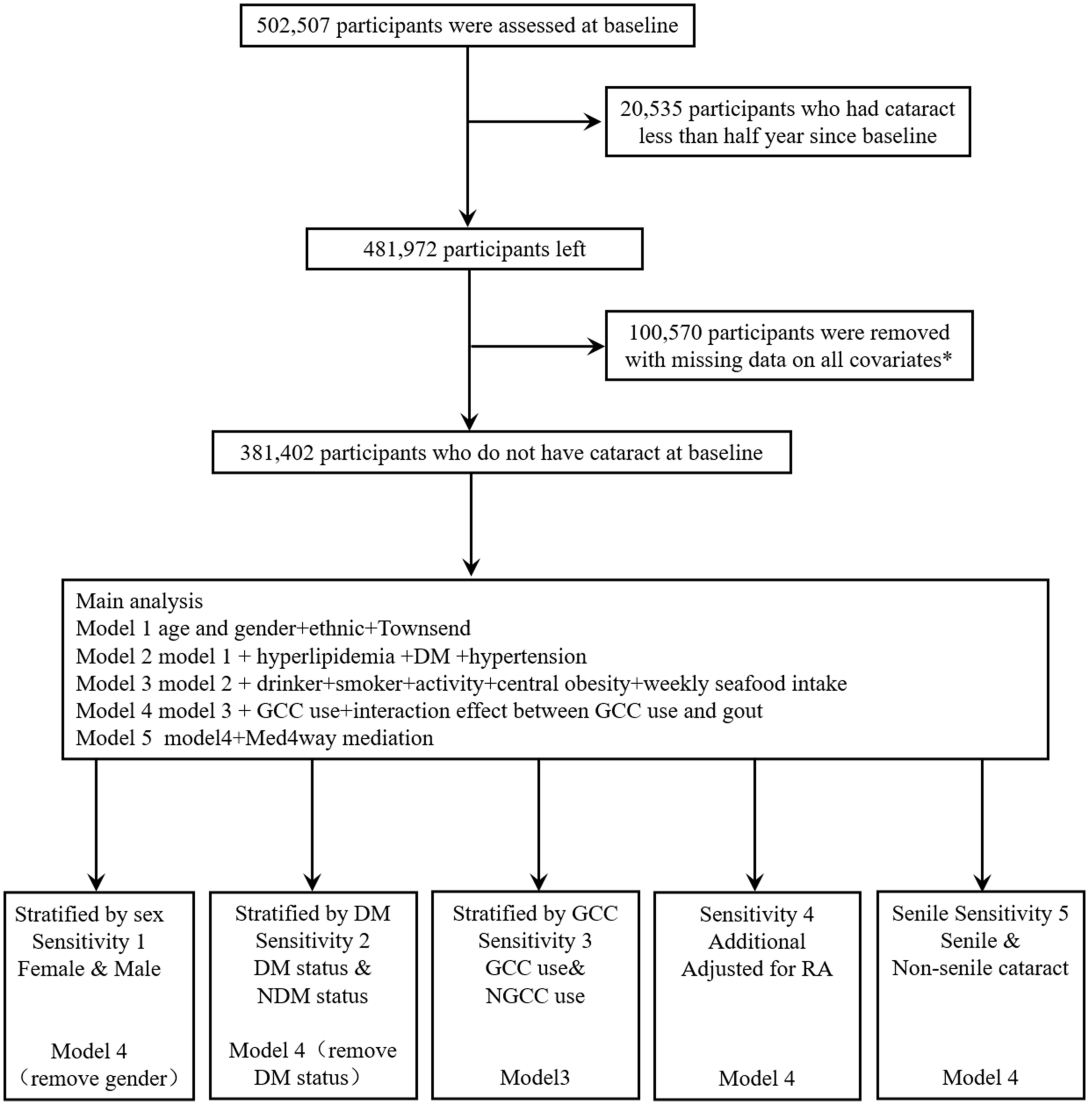
Flowchart of population selection from the UK Biobank. *Covariates: baseline age, gender, ethnic, Townsend deprivation index, Hyperlipidemia status, DM status, hypertension status, alcohol consumption, smoking status, physical activity, central obesity, diet and GCC use.

Cataract diagnosis in the UK Biobank Study was made from hospital inpatient data and self-reported conditions. For hospital admission data, cataract was ascertained by ICD-10 (codes: H25, H262, H263, H264, H268, H269, H280, H281, H282) and ICD-9 (codes: 3661, 3,664, 3,665, 3,668, 3,669). For the self-reported condition, cataract was diagnosed by code 1278. In addition, a questionnaire of ‘Eyesight and Health outcomes’ was also used, and patients who reported having ‘Cataract’ were included as cases. In the sensitivity analysis, senile cataract was defined by ICD-10 (code: H25) and ICD-9 (code: 3661). The earliest recorded diagnosis date was used as the date of cataract onset. Person-years were calculated from the date of baseline assessment to the onset date of cataract, date of death, or the end of follow-up (31 December 2020, for England and Wales and 18 January 2021, for Scotland), whichever is earlier. The selection criteria can be found in Figure 1.

### Covariates

Covariates included baseline age, gender, ethnicity, Townsend deprivation index, hyperlipidemia status, DM status, hypertension status, alcohol consumption, smoking status, physical activity, central obesity, diet, GCC use, and rheumatoid arthritis (RA) status. The ascertainment of covariates is shown in the Supplementary methods.

### Statistical analysis

Data were expressed as frequency (percentage) if categorical and means ± standard deviations if continuous. In comparison of the demographic information, for continuous variables, two-sample T-test was used, while for categorical variables, a chi-squared test was used to estimate the difference between the cataract and non-cataract groups. Cox proportional hazard regression models were then used to examine the association between gout and incident cataract. More details are shown in the Supplementary methods.

In the sensitivity analysis, the association between gout and incident cataract was stratified by sex, DM status, and GCC use. Data analyses were conducted using STATA, and all *p*-values were two-sided with statistical significance set at <0.05.

## Results

### Population selection and baseline cohort descriptions

Figure 1 shows that 502,507 participants were assessed at baseline. We excluded participants who had a cataract less than half a year since baseline (*n* = 20,535), participants with missing data on all covariates at baseline (*n* = 100,570). Finally, 381,402 individuals were enrolled in the analysis. The mean follow-up time was 128.6 months (SD = 24 months) during which time 18,075 (4.74% of the total) participants developed cataract.

Participants with cataract were more likely to be older, male, non-white individuals, smokers, DM patients, hyperlipidemia patients, hypertension patients, central obesity individuals, more fish eaters, and GCC use patients (Table 1). Especially, the prevalence of baseline gout was 1.84% (7,009 individuals) in the final study cohort (non-cataract 1.8%; cataract 2.6%; *p* < 0.001) (Table 1).

**Table 1:** Baseline characteristics of participants according to cataract.

Variables	Non-cataract *n* (%)	Mean (SD)	Cataract *n* (%)	Mean (SD)	*p* value*
Age (years)					<0.001
20–50 *n* = 95,757	95,015 (26.15%)	44.92 ± 2.74	742 (4.10%)	46.06 ± 2.50	
50–60 *n* = 129,848	125,596 (34.57%)	54.70 ± 2.88	4,252 (23.52%)	55.74 ± 2.65	
60–70 *n* = 154,212	141,381 (38.91%)	63.88 ± 2.76	12,831 (71.00%)	64.69 ± 2.83	
≥70 *n* = 1,585	1,335 (0.36%)	70.01 ± 0.11	250 (1.38%)	70 ± 0.00	
Gender					<0.001
Female 0 *n* = 199,986	190,036(52.3%)		9,950 (55.0%)		
Male 1 *n* = 181,416	173,291 (47.7%)		8,125 (45.0%)		
Ethnicity					0.009
Whites *n* = 362,060	344,977 (94.9%)		17,083 (94.5%)		
Other *n* = 19,342	18,350 (5.1%)		992 (5.5%)		
Weekly seafood intake, median (IQR)	4 (2, 4)		4 (3, 4)		<0.001
Above moderate/vigorous/walking	296,501 (81.6%)		14,769 (81.7%)		0.73
Drinker	349,506 (96.2%)		17,146 (94.9%)		<0.001
Smoker	162,934 (44.8%)		8,806 (48.7%)		<0.001
DM status	18,867 (5.2%)		1,781 (9.9%)		<0.001
Hyperlipidemia	160,630 (44.2%)		10,105 (55.9%)		<0.001
Hypertension	258,780 (71.2%)		14,175 (78.4%)		<0.001
Central obesity	175,762 (48.4%)		9,666 (53.5%)		<0.001
GCC use	3,112 (0.9%)		278 (1.5%)		<0.001
Gout	6,531 (1.8%)		478 (2.6%)		<0.001

Data are mean ± standard deviation, or number (%). GCCs, glucocorticoids. *ANOVA was used to test the difference of continuous variables between subgroups of cataract, and *χ*2 for categorical variables.

### Gout and incident cataract

**Figure 2:**
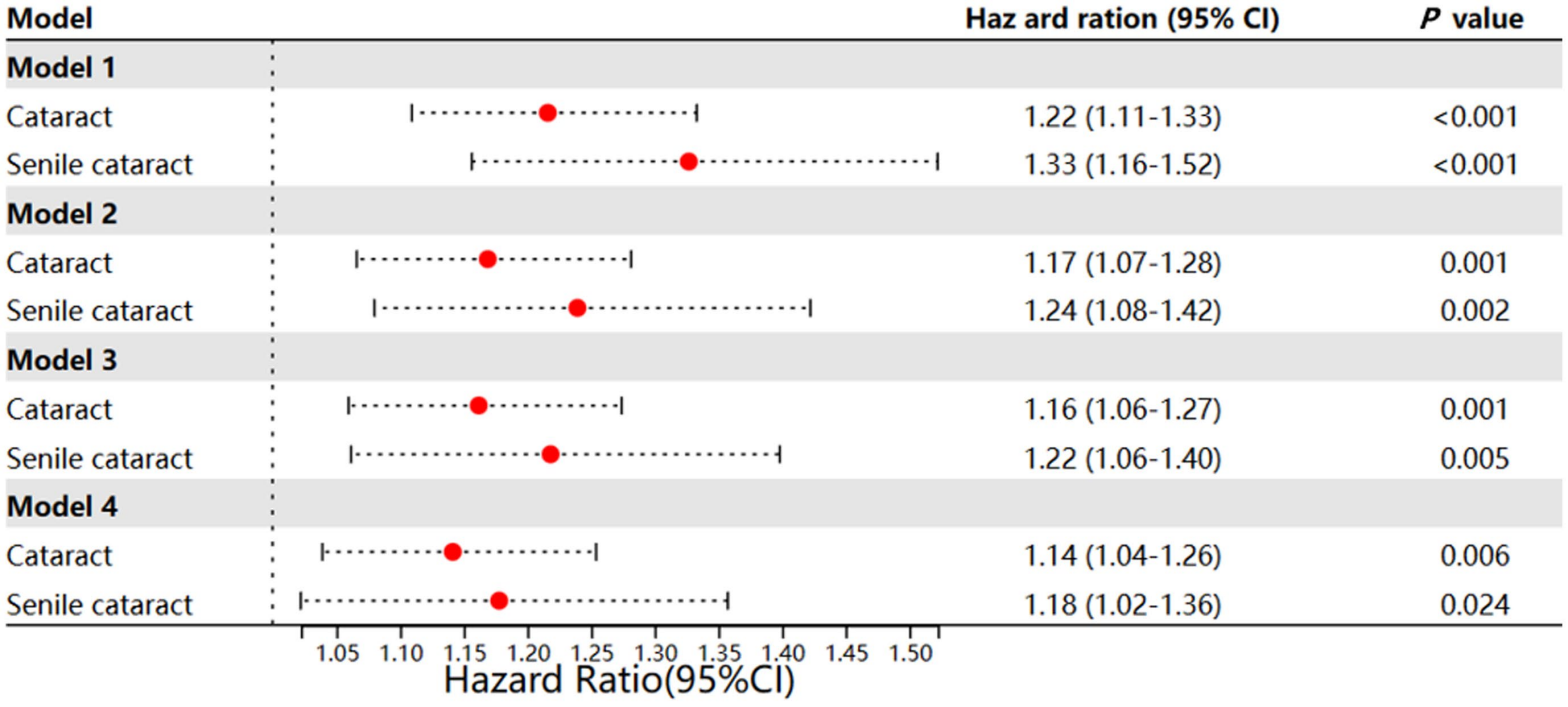
Different models of incident cataract and incident senile cataract. Model 1: Age and gender + ethnic + Townsend; Model 2: model 1 + hyperlipidemia + D + hypertension; Model 3: model 2 + drinker + smoker + activity + central obesity + weekly seafood intake; Model 4: model 3 + GCC use + interaction effect between GCC use and gout.

Gout patients were associated with a higher incidence rate of cataract after adjustment for age, gender, ethnicity, and Townsend deprivation index (HR 1.22; 95% CI, 1.11–1.33; *p* < 0.001; model 1) (Figure 2). This association remained significant after additional adjustment for hyperlipidemia status, DM status, and hypertension status (HR, 1.17; 95% CI, 1.07–1.28; *p* = 0.001; model 2). Moreover, after additional adjustment for lifestyle factors (alcohol use, smoking status, physical activity, central obesity, and weekly seafood intake), the association of gout with incident cataract was slightly reduced but remained significant (HR, 1.16; 95% CI, 1.06–1.27; *p* = 0.001; model 3). When additional considering the use of GCC, gout was found to be associated with a higher incidence of cataract (HR, 1.14; 95% CI, 1.04–1.26; *p* = 0.006; model 4); meanwhile, GCC use was also identified as a risk factor for cataract (HR = 1.42; 95% CI, 1.25–1.60; *p* < 0.001; model 4).

As senile cataract was more associated with metabolic disease, we further investigated the incidence of senile cataract in gout patients. Similar findings were observed: adjusting for baseline information, lifestyle, and GCC use step-wisely could not eliminate the effect of gout on the incidence of senile cataract development (Figure 2; Supplementary Tables 1–4). Different from cataract, in model 4, we observed a significant interaction effect between GCC use and gout (HR, 1.85, 95% CI, 1.03–3.33; *p* = 0.040), this might indicate that gout might have greater impact on senile cataract (four association models, model 1 HR, 1.33, 95% CI, 1.16–1.52, *p* < 0.001; model 2 HR, 1.24, 95% CI, 1.08–1.42, *p* = 0.002; model 3 HR, 1.22, 95% CI, 1.06–1.40, *p* = 0.005; model 4 HR, 1.18, 95% CI, 1.02–1.36, *p* = 0.024.)

### Mediating effects of GCC on the association between gout and time to cataract

Before we conducted the mediation analysis, we identified that, compared to patients without gout, patients with gout had an increased risk of being prescribed GCC (OR = 2.13, 95% CI: 1.82–2.50, *p* < 0.001). We also identified that compared to patients who were not taking GCC, patients taking GCC had an HR of 1.42 (95% CI: 1.25–1.60) for developing cataract. These findings support the validity of using GCC as the mediator. We then estimate the mediation and interaction effect of GCC. In the Cox models, having gout and GCC use were both associated with shorter time to developing cataract as well as senile cataract [For cataract: HR_gout = 1.14 (95%CI: 1.04–1.25), HR_GCC = 1.42 (95%CI: 1.25–1.60); For senile cataract: HR_gout = 1.18 (95%CI: 1.02–1.36), HR_GCC = 1.68 (95% CI: 1.41–2.00)]. The mediator model also found that prior gout was positively associated with GCC use (for cataract OR = 2.13, *p* < 0.001; for senile cataract OR = 2.13, *p* < 0.001).

The mediation decomposition indicated that the total effect was driven by both direct effects of gout and indirect effects via GCC, while there was a mixed mediation effect. The total excess risk is then decomposed into four parts: we found that the total, controlled direct, and pure indirect effect of gout were significant in the cataract (*p*_total = 0.003, *p_*direct = 0.010, and *p_*indirect < 0.001), and GCC use mediated around 2.4% of the effect of gout on cataract (*p* = 0.018). However, the reference interaction and mediated interaction were not significant in cataract (*p* = 0.110, *p* = 0.120) (Table 2, model 5). These findings indicate the important role of GCC in the association between gout and cataract.

**Table 2:** Med4way (model 5 for cataract and senile cataract).

Component	Cataract				Senile cataract			
	Coefficient	95%CI		*p*	Coefficient	95%CI		*p*
Gout	1.142	1.039	1.254	0.006	1.178	1.022	1.358	0.024
GCC	1.419	1.254	1.604	<0.001	1.679	1.410	2.001	<0.001
Gout × GCC	1.514	0.952	2.409	0.080	1.852	1.029	3.333	0.040
Mediator logistic								
Gout	2.133	1.824	2.496	<0.001	2.133	1.824	2.496	<0.001
4-Way decomposition								
Total excess relative risk	0.161	0.054	0.268	0.003	0.215	0.048	0.383	0.012
Controlled direct effect	0.141	0.034	0.248	0.010	0.177	0.011	0.343	0.037
Reference interaction	0.007	−0.002	0.017	0.110	0.015	−0.002	0.032	0.080
Mediated interaction	0.008	−0.002	0.019	0.120	0.017	−0.003	0.036	0.090
Pure indirect effect	0.004	0.002	0.006	<0.001	0.006	0.003	0.010	<0.001

The same analysis was conducted for senile cataract. After confirming, the GCC use as a validate mediator, we note that, the total excess risk is then decomposed into four parts: we found that the total, controlled direct and pure indirect effect of gout were significant in the cataract (*p*_total = 0.012, *p_*direct = 0.037 and *p_*indirect < 0.001), and GCC use mediated approximately 3% of the effect of gout on senile cataract (*p* = 0.034). However, the reference interaction and mediated interaction were not significant in the cataract (*p* = 0.08, *p* = 0.09) (Table 2, model 5).

### Gout and incident cataract stratified by gender, DM status, and GCC use

**Figure 3:**
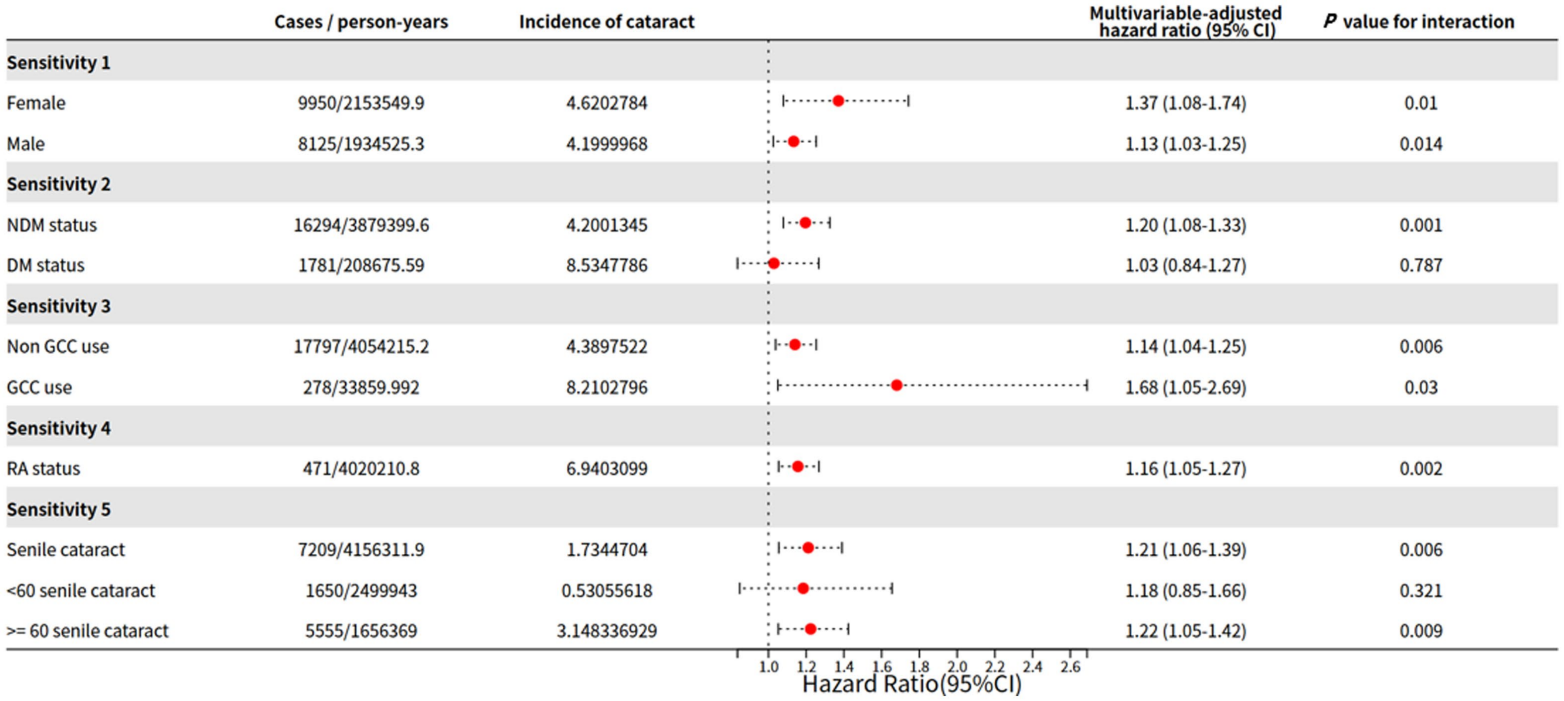
Sensitivity analysis of gender, DM, GCC use, RA status, and age.

In the first sensitivity analysis, we stratified the data by gender to analyze the impact of gout on cataract in different genders, as females were reported to be more likely to develop cataract [9, 25]. As shown in Figure 3, consistent with previous studies, the incidence of cataract in females was 4.62%, whereas that of males was 4.20%. The effect of gout on the incidence of cataract was both significant in females (HR, 1.37; 95% CI, 1.08–1.74; *p* = 0.010) and males (HR, 1.13; 95% CI, 1.03–1.25; *p* = 0.014) (Figure 3; Supplementary Tables 5, 6).

Then we stratified the data by DM status to further determine the association between DM status and gout for incident cataract. Similarly, the incidence of cataract in individuals with DM status was 8.53%, whereas that of individuals with NDM status was 4.20%. The effect of gout on the incidence of cataract was significant for participants without DM (HR, 1.20; 95% CI, 1.08–1.33; *p* = 0.001). However, having gout did not increase the risk of incident cataract in participants who were diagnosed with DM (HR, 1.03; 95% CI, 0.84–1.27; *p* = 0.787). This might be attributed to the high incidence of cataract in diabetic patients themselves (Figure 3; Supplementary Tables 7, 8).

GCC, one type of first-line medication for gout patients, could favor cataract formation [9, 26]. The associations between gout and incident cataract were both positive and significant in individuals with non-GCC use (HR, 1.14; 95% CI, 1.04–1.25; *p* = 0.006) or GCC use (HR, 1.68; 95% CI, 1.05–2.69; *p* = 0.030) (Figure 3; Supplementary Tables 9, 10).

### Gout and incident cataract additional adjust for rheumatoid arthritis

Since the UK Biobank does not provide information on whether GCC use was specifically for gout, and given that GCCs are also a primary treatment for RA, we conducted a further sensitivity analysis (sensitivity analysis 4) focusing on RA. As shown in Figure 3, gout remained associated with a higher incidence of cataract after adjustment for RA (HR = 1.16, 95% CI: 1.05–1.28, *p* = 0.002) (Figure 3; Supplementary Table 11). Moreover, the mediator model also found that prior gout was positively associated with GCC use after adjusting for RA (OR = 2.014, *p* < 0.001) (Supplementary Table 12).

### Gout and incidence of senile cataract

Then we focus on the effect of gout on senile cataract. Similar to the overall analysis, we found that gout patient was also associated with a 1.73% incidence rate of senile cataract after adjustment for the covariates than non-gout patients (HR, 1.21; 95% CI, 1.06–1.39; *p* = 0.006). Specifically, the incidence of senile cataract in gout patients ≥60 years of age is significant, whereas that in patients <60 years of age was insignificant (<60 gout patients: incidence of cataract 0.53%, HR, 1.18; 95% CI, 0.85–1.66, *p* = 0.321; ≥60 gout patients: incidence of cataract 3.15%, HR, 1.22; 95% CI, 1.05–1.42, *p* = 0.009) (Figure 3; Supplementary Tables 13–15).

## Discussion

This large-scale prospective study, conducted by enrolling participants from the UK Biobank, aimed to investigate the correlation between gout and the development of cataract. The findings revealed a significant association between gout and an increased risk of cataract incidence. Notably, gout patients who used GCC exhibited a higher incidence rate of developing cataract. Furthermore, after adjusting for age, gender, and ethnicity, which are established risk factors for age-related cataract, gout was still associated with incident cataract and senile cataract. The analysis showed hazard ratios of 1.14 for cataract and 1.18 for senile cataract, indicating a notable correlation between gout and the development of both cataract types. Additionally, this study investigated the role of GCC use and identified both an interaction and a mediation effect of GCC on the association between gout and cataract. These findings suggest that long-term exposure to uric acid, particularly in older adults, may increase the risk of cataract formation.

Previous observational studies have reported associations between gout and various types of cataracts, such as nuclear, posterior subcapsular, and cortical cataracts [27]. However, these studies were limited in design, using a cross-sectional approach and involving relatively small sample sizes. In contrast, our study enrolled a large cohort of 381,402 patients, making it the most extensive study conducted thus far, utilizing a cohort study design. Even after adjusting for potential confounding factors, gout patients consistently demonstrated a strong association with incident cataract compared to non-gout patients. This association persisted when examining senile cataract and when stratifying the population based on sex, DM status, and GCC use, further affirming a robust relationship between gout and incident cataract.

The underlying biological mechanism linking gout and cataract is likely to be intricate. Prior reports have suggested that elevated serum urate deposition may increase the risk of PSC (OR = 1.14) [28, 29]. Our previous clinical study and experimental investigation support this finding by demonstrating a positive correlation between increased serum uric acid levels and elevated uric acid levels in the aqueous humor [30]. Specifically, a significant accumulation of urates was observed in the equatorial lens fiber cells and posterior capsule of patients with PSC. Moreover, we found that uric acid induced the incidence of cataract mainly through the NLRP3/caspase-1/IL-1β signaling pathway [31]. Additionally, a retrospective comparative study indicated that gout patients who underwent cataract surgery were more susceptible to corneal endothelial cell injury compared to patients without gout [32]. Both uric acid levels and duration of gout, which might be attributed to the decreased activity of Na, K-ATPase [32, 33], indicating that the duration of uric acid could alter the eye metabolism.

Furthermore, the use of gout medication, such as colchicine, was found to accelerate the progression of lens opacification, particularly in gout patients aged over 60 years who received higher cumulative doses of colchicine [17]. In our study, we also investigated the relationship between gout, GCC use, and cataract while considering the comprehensive role of GCC. According to recent guidelines, GCC is a frontline treatment for gout and is a strong risk factor for PSC [9, 26, 34]. GCC could inhibit the sodium–potassium pumps in the lens epithelium, induce changes in the transcription of genes in lens epithelial cells, accumulate water in lens fibers, and agglutinate the lens protein [35, 36]. The duration and dosage of GCC use are positively associated with cataract formation.

Our study included the consideration of GCC use and explored both the interaction effect of gout on cataract development and the mediation effect on the relationship between gout and cataract. Consistent with previous research, our results confirm that GCC use is associated with an increased risk of cataract. Moreover, we observed a positive interaction effect between gout and GCC use. This suggests a synergistic effect where the combined presence of gout and GCC use confers a risk exceeding what would be expected from their individual contributions. It is crucial to note, however, that our mediation analysis indicated GCC use explained only a very small fraction (2.4% for cataract and 3% for senile cataract) of the total association between gout and cataract. Therefore, while statistically significant, the clinical contribution of this mediation pathway through GCC appears limited. The vast majority of the excess risk is likely attributable to the core pathological mechanisms of gout itself, such as chronic hyperuricemia, systemic oxidative stress, and inflammation. These findings underscore that for gout patients, especially those requiring GCC therapy, there exists a synergistic risk profile for cataract development.

Subsequently, we conducted stratified analyses based on age, gender, diabetes, and GCC use, as these factors are key risk factors for cataract development. These analyses demonstrated that gout had a significant impact on incident cataract in women, patients without diabetes, and patients using GCC (Figure 3). Females were more likely to have cataract compared to males and when we stratified the population by gender, we noticed that gout increased the risk of cataract in both genders. It was found that the HR of gout on cataract in females was greater than in males. We suspect this might be due to the reason that the decrease in estrogen at menopause causes an increased risk of cataract in women, not strictly the concentration of estrogen, but more the withdrawal effect [37]. Prior studies have highlighted the importance of diabetes in cataract development [9, 38–40]. Our previous research has shown the significant role of diabetes in the connection between obesity and incident cataract [22]. In our stratified analysis, diabetes patients exhibited the highest incidence of cataract (incidence rate: 8.53%), while the influence of gout on cataract in this group was insignificant (HR: 1.03; 95%CI: 0.84–1.27; *p* = 0.787). Among those using GCC, the second-highest incidence of cataract was observed (incidence rate: 8.21%), and in this group, gout posed the highest risk of cataract (HR: 1.68; 95% CI: 1.05–2.69; *p* = 0.030). Additionally, gout patients over the age of 60 demonstrated a higher susceptibility to senile cataracts with a HR 1.22 (95%CI: 1.05–1.42; *p* = 0.009). These findings can guide elderly gout patients to pay closer attention to the use of gout medication and the development of cataracts.

To date, this study is the first and largest cohort study comprehensively investigating the association between gout and incident cataract. We innovatively investigated the relationship between gout, GCC use, and cataract, and explored the differential effects of gout on incident cataract and senile cataract. However, there are limitations to our study. First, the outcome assessment was restricted to broad cataract categories, lacking subtype details that could offer more specific biological insights. Second, detailed data on glucocorticoid use (dosage, duration, frequency) were unavailable, precluding a refined exposure-response analysis. Third, our findings are derived solely from the UK Biobank and require external validation in independent, ethnically diverse cohorts, which may limit generalizability. Fourth, the use of a standard Cox model, which estimates hazards among individuals under active follow-up, does not formally account for competing mortality risks. As gout patients have a higher background mortality rate, this could lead to an underestimation of their true cumulative incidence of cataract. Finally, residual confounding from factors such as fluctuating uric acid levels cannot be ruled out.

In conclusion, our study provides comprehensive evidence on the relationship between gout, GCC use, and cataract. We discovered that gout is an independent risk factor for cataract, and GCC use partially mediates its effect on cataract while additionally contributing to cataract progression through an interaction effect. Therefore, caution should be exercised in medication management for gout patients, especially regarding the use of GCC.

## Data Availability

The raw data supporting the conclusions of this article will be made available by the authors, without undue reservation.

## Supplementary material

SUPPLEMENTARY FIGURE 1 Directed acyclic graph for the relationship between gout and cataract, and mediation through GCC use. Mediation effects are decomposed into controlled direct effects (through arrow c), reference interaction (through arrows c and d), mediated interaction (through arrows a, c, and d), and pure indirect effects (through arrows a and b).
