# Supplementary figures and images for: Assessing the relationship between gout and the risk of cataract in community-dwelling older adults: mediation and moderation analysis
Source: Front Med (Lausanne). 2026 Jan 13;12:1740517. doi: 10.3389/fmed.2025.1740517 (PMC12836401; doi:10.3389/fmed.2025.1740517)

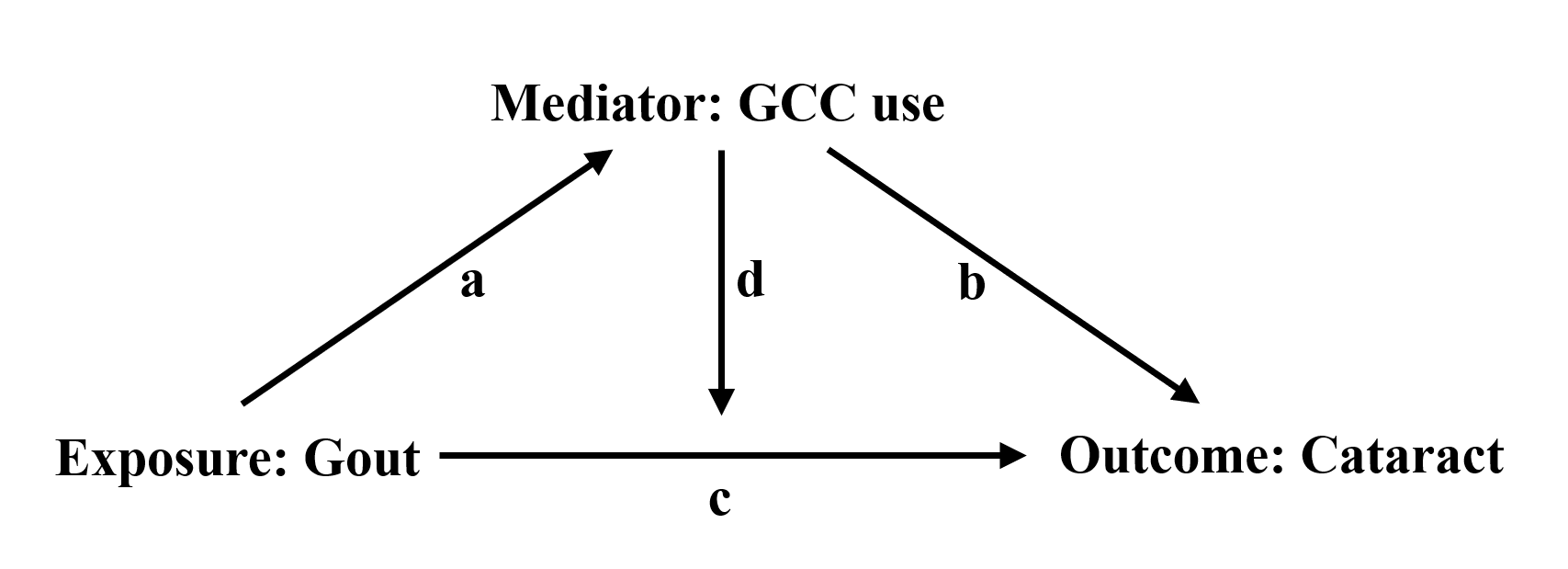

Supplement: SUPPLEMENTARY FIGURE 1 — Directed acyclic graph for the relationship between gout and cataract, and mediation through GCC use. Mediation effects are decomposed into controlled direct effects (through arrow c), reference interaction (through arrows c and d), mediated interaction (through arrows a, c, and d), and pure indirect effects (through arrows a and b). [file Image_1.TIF]
