# Supplementary material for: Assessing the relationship between gout and the risk of cataract in community-dwelling older adults: mediation and moderation analysis
Source: Front Med (Lausanne). 2026 Jan 13;12:1740517. doi: 10.3389/fmed.2025.1740517 (PMC12836401; doi:10.3389/fmed.2025.1740517)
Supplement: Supplementary file 2 [file Table_2.DOCX]

Supplementary method: the data-field (codes) of covariates in UK biobank.

**Baseline age:** 21022.

**Gender:** 31.

**Ethnic:** 21000.

**Townsend deprivation index:** 189.

Hyperlipidemia status: 20009, 6153, 30690, 20003 (1141146234, 1141146138, 1140888594, 1140864592, 1140888648, 1140861970, 1141192410, 1141192414, 1140861958, 1140881748, 1141188146, 1141192736, 1141192740, 1140861924, 1141201306, 1140861926, 1140861928, 1140862026, 1140861954, 1141162544, 1141172214, 1140861856, 1141157262, 1140861858, 1140861892, 1140861894, 1140861868, 1141188546).

**DM status,：**2443, 6153, 20003, 30750 (1140883066, 1140884600, 1140874686, 1141189090, 1140874718, 1140874744, 1140874746, 1141152590, 1141156984, 1140874646, 1141157284, 1140874652, 1140874674, 1140874728, 1140868902, 1140868908, 1140857508, 1141173882, 1141173786, 1141168660, 1141171646, 1141171652, 1141153254, 1141177600, 1141177606).

**Hypertension status:** 6153, 20009, 20002 (1065, 1072), 4079, 4080, 20003 (1140860192, 1140860292, 1140860308, 1140860312, 1140860316, 1140860320, 1140860322, 1140860332, 1140860336, 1140860340, 1140860342, 1140860404, 1140860418, 1140860422, 1140860426, 1140860470, 1140860562, 1140860696, 1140860728, 1140860738, 1140860750, 1140860764, 1140860790, 1140860806, 1140860904, 1140861088, 1140861190, 1140864202, 1140864950, 1140864952, 1140866078, 1140866090, 1140866102, 1140866108, 1140866138, 1140866144, 1140866162, 1140866200, 1140866206, 1140866236, 1140866280, 1140866324, 1140866330, 1140866332, 1140866388, 1140866422, 1140866426, 1140866448, 1140866724, 1140866738, 1140871986, 1140872568, 1140875840, 1140875934, 1140879658, 1140879758, 1140879760, 1140879778, 1140879782, 1140879786, 1140879794, 1140879798, 1140879802, 1140879806, 1140879810, 1140879818, 1140879822, 1140879824, 1140879830, 1140879834, 1140879842, 1140879866, 1140883468, 1140888510, 1140888512, 1140888552, 1140888556, 1140888560, 1140888646, 1140888686, 1140909368, 1140909708, 1140910358, 1140910606, 1140916356, 1140923712, 1140926778, 1140928226, 1140928284, 1141145660, 1141146124, 1141146126, 1141146128, 1141151016, 1141152998, 1141153026, 1141153328, 1141156836, 1141157184, 1141157490, 1141164276, 1141165470, 1141166006, 1141169516, 1141171336, 1141172682, 1141180592, 1141180772, 1141180778, 1141184722, 1141186674, 1141187788, 1141193282, 1141194794, 1141194800, 1141194804, 1141194808, 1141194810, 1141195254, 1141195258, 1141201038, 1141201244).

**Alcohol consumption:** 20117.

**Smoking status:** 20116.

**Physical activity:** 22032.

**Central obesity:** 21001, 48, 49.

**Diet:** 1329, 1339.

**GCC use:** 20003 (1140868364, 1140874976, 1140874790, 1140874930, 1140874896, 1141173346, 1140884704, 1140874816).

**RA status:** ICD-10 (41270, 410202, 41204: M05.0, M05.1, M05.2, M05.3, M05.8, M05.9, M06.0, M06.1, M06.2, M06.3, M06.4, M06.9), ICD-9 (41271, 41203, 41205: 7140), self-report (20002: 1464).
